# 2G ethanol from the whole sugarcane lignocellulosic biomass

**DOI:** 10.1186/s13068-015-0224-0

**Published:** 2015-03-12

**Authors:** Sandra Cerqueira Pereira, Larissa Maehara, Cristina Maria Monteiro Machado, Cristiane Sanchez Farinas

**Affiliations:** Embrapa Instrumentation, Rua XV de Novembro 1452, 13560-970 São Carlos, SP Brazil; Graduate Program of Chemical Engineering, Federal University of São Carlos, Rodovia Washington Luiz, km 235, 13565-905 São Carlos, SP Brazil; Embrapa Agroenergy, Parque Estação Biológica s/n°, 70770-901 Brasília, DF Brazil

**Keywords:** Sugarcane bagasse, Sugarcane trash, Second-generation ethanol, Whole sugarcane lignocellulosic biomass, Cellulosic ethanol, Bioethanol

## Abstract

**Background:**

In the sugarcane industry, large amounts of lignocellulosic residues are generated, which includes bagasse, straw, and tops. The use of the whole sugarcane lignocellulosic biomass for the production of second-generation (2G) ethanol can be a potential alternative to contribute to the economic viability of this process. Here, we conducted a systematic comparative study of the use of the lignocellulosic residues from the whole sugarcane lignocellulosic biomass (bagasse, straw, and tops) from commercial sugarcane varieties for the production of 2G ethanol. In addition, the feasibility of using a mixture of these residues from a selected variety was also investigated.

**Results:**

The materials were pretreated with dilute acid and hydrolyzed with a commercial enzymatic preparation, after which the hydrolysates were fermented using an industrial strain of *Saccharomyces cerevisiae*. The susceptibility to enzymatic saccharification was higher for the tops, followed by straw and bagasse. Interestingly, the fermentability of the hydrolysates showed a different profile, with straw achieving the highest ethanol yields, followed by tops and bagasse. Using a mixture of the different sugarcane parts (bagasse-straw-tops, 1:1:1, in a dry-weight basis), it was possible to achieve a 55% higher enzymatic conversion and a 25% higher ethanol yield, compared to use of the bagasse alone. For the four commercial sugarcane varieties evaluated using the same experimental set of conditions, it was found that the variety of sugarcane was not a significant factor in the 2G ethanol production process.

**Conclusions:**

Assessment of use of the whole lignocellulosic sugarcane biomass clearly showed that 2G ethanol production could be significantly improved by the combined use of bagasse, straw, and tops, when compared to the use of bagasse alone. The lower susceptibility to saccharification of sugarcane bagasse, as well as the lower fermentability of its hydrolysates, can be compensated by using it in combination with straw and tops (sugarcane trash). Furthermore, given that the variety was not a significant factor for the 2G ethanol production process within the four commercial sugarcane varieties evaluated here, agronomic features such as higher productivity and tolerance of soil and climate variations can be used as the criteria for variety selection.

## Background

The growing concern over the shortage of oil reserves, together with the need to preserve the environment, is the main drivers of the search for viable alternative renewable sources for the production of sustainable fuels. Furthermore, since the transportation sector accounts for about 16% of global greenhouse gas (GHG) emissions [1] and about 27% of total US GHG emissions [2], the replacement of oil-derived fuels by biofuels could contribute to the reduction of environmental impacts as well as provide socio-economic benefits [3,4]. There is already a consensus that a shift of the global energy scenario towards increasing the share of renewable sources is necessary and that ethanol will undoubtedly be an important biofuel [5]. The need to produce inexpensive renewable fuels to substitute fossil fuels is revealed in the political agendas of several countries [6]. Therefore, the future energy economy is likely to be based on a wide range of alternative energy platforms. Among the potential bioenergy sources, the lignocellulosic biomass has been identified as an important feedstock for the production of biofuels and other value-added products, thus contributing to the global energy supply. In this context, Brazil is notable in that the country has made considerable progress towards replacing fossil fuels by renewable ethanol from sugarcane. Moreover, Brazil is the world’s largest producer of sugarcane and together with US leads the global production of bioethanol [7-9]. In the 2014/15 harvest, it is estimated that more than 640 million tons of sugarcane will be processed by the Brazilian sugar-alcohol mills, which will result in an ethanol production of approximately 28 billion liters and a sugar production of about 36 million tons [7].

In the sugarcane industry, large amounts of lignocellulosic residues (bagasse and trash) are generated during the production of ethanol and sugar. After the sugarcane harvest, the trash remaining consists of three main components, namely, dry leaves, green leaves, and tops [10]. The tops are the segments of the sugarcane plant between the upper end and the last stalk node with attached green leaves, while the dry leaves are known as straw. The milling of the sugarcane to extract the juice generates the bagasse. In this process, every ton of sugarcane processed generates 140 kg of bagasse and 140 kg of trash, on a dry basis (db) [11]. In contemporary facilities, the bagasse is currently used as fuel, that is, it is burned in boilers to meet the energy demands of the industrial plant, with the surplus electric energy being exported to the grid [12-14]. The sugarcane trash was previously burned prior to the harvest in order to facilitate the harvest procedure, but is now mostly left in the field for agricultural purposes as fertilizer and for pest control [14]. There is currently an ongoing progressive shift in the sugarcane harvesting method, from manual harvesting of burned sugarcane to mechanical harvesting of unburned sugarcane, with the trash remaining on the ground [14-16]. This increasing amount of lignocellulosic material being left on the ground could be partially recovered and used for energy generation in the mills, thus improving the overall energy balance [14].

The second-generation (2G) ethanol produced from lignocellulosic biomass has been considered to be the biofuel with the greatest potential to replace oil-based fuels [17,18]. However, 2G technology is not as mature as that of conventional first-generation (1G) ethanol, and the process is still less economically feasible [12,19]. In energy terms, it is known that the energy content (MJ/kg) of sugarcane trash is similar to that of the bagasse, with the two components accounting for approximately two thirds of the total energy content of sugarcane [11,15]. Despite the fact that the trash is currently considered the main unexploited energy reserve in the sugarcane agro-industry, its most likely use in the short to medium term will be as boiler fuel [11,15]. Nonetheless, considering the amounts of residues generated and that all the cellulose present in these materials could be converted into ethanol, the use of the whole sugarcane lignocellulosic biomass (including trash and bagasse) could greatly increase ethanol production without the need for expansion of cultivated areas. It is worth mentioning that there is still no consensus about the optimal amount of trash that should be left on the ground. It has been observed that 65% of the residue from sugarcane harvesting can be removed from the fields as dry leaves (straw), without causing negative agricultural effects [20].

There have been several studies of 2G ethanol production processes using sugarcane bagasse [21-29]. In one study, evaluation was made of the bagasse from 115 varieties of sugarcane developed by classical and precision breeding technologies, in terms of fiber composition and fermentable sugar yields after dilute acid pretreatment and enzymatic hydrolysis [21]. It was suggested that variety selection could contribute to the development of a cost-effective pretreatment and saccharification process. After the screening reported previously [21], the authors also described the optimization of pretreatment of selected varieties, used in the cellulosic ethanol production process in order to fully demonstrate the benefits of variety selection in terms of the yields of fermentable sugar and ethanol from the bagasse [23,24].

On the other hand, there have been few reports concerning the individual assessment of other parts of the sugarcane biomass (straw and tops) for the development of 2G ethanol production processes [30-32] or the combined use of lignocellulosic sugarcane residues [33,34]. In fact, the combined use of sugarcane residues has been investigated only regarding the enzymatic hydrolysis step, employing either hydrothermal [33] or dilute acid [34] pretreated materials. Nevertheless, to the best of our knowledge, there have not been any systematic studies of 2G ethanol production using the whole lignocellulosic biomass from different commercial sugarcane varieties with known agronomic advantages in terms of productivity as well as tolerance of soil and climate variability.

The aim of the present study was to contribute to the development of 2G ethanol production processes by evaluating a biotechnological approach employing the whole sugarcane lignocellulosic biomass. The lignocellulosic residues (bagasse, straw, and tops) from four commercial sugarcane varieties (SP79-1011, RB867515, SP81-3250, and RB92579) were assessed for 2G ethanol production, with determination of the individual responses obtained for different process stages (dilute sulfuric acid pretreatment, enzymatic hydrolysis, and alcoholic fermentation). The parameters considered were chemical composition, susceptibility to saccharification, and fermentability. Investigation was also carried out on the combined use of a mixture of bagasse, straw, and tops, under the same conditions.

## Results and discussion

### Chemical composition of the raw material

Three lignocellulosic residue parts (bagasse, straw, and tops) from the processing of four commercial sugarcane varieties (SP79-1011, RB867515, SP81-3250, and RB92579, represented here by K, M, Q, and X, respectively) were evaluated according to their chemical composition in terms of hemicellulose, lignin, cellulose, and ash contents (*w*/*w*, dry weight basis) (Table 1). As can be seen, the lignocellulosic residue parts differed considerably in their chemical composition. Overall, cellulose and hemicellulose were the main components of the biomass, accounting for 34.1 to 42.1% and 28.5 to 38.8% of the dry material, respectively. Lignin accounted for 5.6 to 13.8%, and the ash content of the raw material was in the range 0.8 to 3.8%.

**Table 1:** **Chemical composition of the three lignocellulosic residue parts (straw, tops, and bagasse)**

**Variety**	**Symbol**	**Residue**	**Cellulose**	**Hemicellulose**	**Lignin**	**Ash**
SP79-1011	K	Straw	41.09	35.07	11.13	2.25
		Tops	34.68	35.92	8.00	3.63
		Bagasse	36.60	28.45	13.81	0.80
RB867515	M	Straw	42.12	35.93	12.04	2.81
		Tops	35.02	37.27	8.95	3.65
		Bagasse	38.47	28.86	13.45	0.79
SP81-3250	Q	Straw	41.27	36.53	11.33	1.72
		Tops	34.05	38.79	9.08	3.82
		Bagasse	36.13	29.19	12.83	1.14
RB92579	X	Straw	39.91	37.07	11.26	2.07
		Tops	37.08	38.76	7.59	3.30
		Bagasse	39.07	31.02	12.89	0.88

Data refer to the four varieties of sugarcane (K, M, Q, and X), in terms of cellulose, hemicellulose, lignin, and ash contents (*w*/*w*, dry weight basis). Data are means of three replicates.

For all the varieties, the highest cellulose content was found for the straw, followed by the bagasse and tops (Table 1). On the other hand, all the varieties showed the highest levels of hemicellulose in the tops, followed by the straw and bagasse. For all the varieties, the lignin content was highest in the bagasse, followed by the straw and tops, while the ash content was highest in the tops, followed by the straw and bagasse. It can therefore be seen that determination of chemical composition is vital because the conversion of vegetal biomass into biofuel (by means of the sequential stages of pretreatment, enzymatic hydrolysis, and alcoholic fermentation) is closely connected to the inherent features of these lignocellulosic materials. Overall, the raw materials showed similar chemical composition profiles, irrespective of the variety.

The overall average cellulose, hemicellulose, lignin, and ash contents of the untreated straw from the four varieties of sugarcane were 41.1 ± 0.9, 36.2 ± 0.9, 11.4 ± 0.4, and 2.2 ± 0.5%, respectively (Table 1). These values differ from those described in the literature, mainly because the majority of studies were carried out with sugarcane trash as the raw material and not with straw alone. For example, in one study, the contents of cellulose, hemicellulose, lignin, and ash were 33.6, 28.9, 31.8, and 5.7%, respectively [35], while other work reported values of 39.8, 28.6, 22.5, and 2.4%, respectively [30]. Moreover, the average contents of cellulose, hemicellulose, lignin, and ash found for the untreated bagasse from the four varieties of sugarcane were 37.6 ± 1.4, 29.4 ± 1.1, 13.2 ± 0.5, and 0.9 ± 0.2%, respectively, and differed only slightly from the values reported in the literature [21,25-27,34,36]. Few studies have evaluated the chemical composition of the tops separately. For the four varieties, the average contents of cellulose, hemicellulose, lignin, and ash in the tops were 35.2 ± 1.3, 37.7 ± 1.4, 8.4 ± 0.7, and 3.6 ± 0.2%, respectively. In the case of cellulose and hemicellulose (but not lignin and ash), the contents found here for the tops were comparable to those reported in the literature [32,37].

These differences in chemical composition for the same type of biomass were expected, due to the influence of numerous factors including plant variety, growth environment, and processing conditions, as well as the procedures employed for the compositional analysis [38]. Furthermore, it is difficult to compare the compositions of samples from different origins when the analyses are performed by laboratories that do not use the same methods [10]. Nevertheless, in terms of the contents of cellulose, hemicellulose, lignin, and ash, the sugarcane characterization performed here resulted in a range of values similar to those found for lignocellulosic feedstocks in general [39].

Many sugarcane varieties are available in Brazil, which is highly advantageous for the profitability of the sugar-alcohol sector because it enables the selection of varieties that are better adapted to adverse conditions of soil and weather, more resistant to pests and diseases, and suitable for specific harvesting systems. The commercial sugarcane varieties evaluated in this study (SP79-1011, RB867515, SP81-3250, and RB92579) were selected because they are among the most widely used in Brazilian sugarcane plantations, due to their good agronomic characteristics including low demand for water, suitability for mechanical harvesting, and good transportation efficiency, among others.

### Effect of dilute sulfuric acid pretreatment on the feedstock

Table 2 shows the chemical composition of the biomass obtained after dilute acid pretreatment (H_2_SO_4_, 1.5% *w*/*w*, 1:10 solid/liquid ratio, 121°C, 30 min) of the three residue parts (bagasse, straw, and tops) obtained from processing of the four varieties of sugarcane (here denoted K, M, Q, and X). There were only slight differences in the chemical compositions of the pretreated materials. Cellulose was the main component, followed by lignin, while hemicellulose and ash accounted for much smaller proportions of the dry samples. Overall, the contents of cellulose, hemicellulose, lignin, and ash were in the ranges 47.0 to 51.9, 6.2 to 7.9, 29.6 to 32.9, and 0.5 to 1.9%, respectively.

**Table 2:** **Chemical composition of the three pretreated residue parts (straw, tops, and bagasse)**

**Variety**	**Symbol**	**Residue**	**Cellulose**	**Hemicellulose**	**Lignin**	**Ash**
SP79-1011	K	Straw	48.06	6.37	31.82	0.89
		Tops	51.34	7.54	29.62	1.75
		Bagasse	49.37	7.69	30.37	0.51
RB867515	M	Straw	47.44	6.38	32.32	0.99
		Tops	49.18	7.73	30.15	1.84
		Bagasse	47.98	7.88	30.85	0.59
SP81-3250	Q	Straw	47.03	6.89	32.91	1.01
		Tops	48.87	7.26	31.41	1.86
		Bagasse	47.72	7.54	32.48	0.66
RB92579	X	Straw	49.21	6.18	32.94	0.92
		Tops	51.88	7.54	30.37	1.76
		Bagasse	50.50	7.77	31.99	0.52

Data refer to the four varieties of sugarcane (K, M, Q, and X), in terms of cellulose, hemicellulose, lignin, and ash contents (*w*/*w*, dry weight basis). Data are means of three replicates.

As also found for the raw materials (Table 1), the chemical composition profiles of the pretreated residues (bagasse, straw, and tops) were similar for the four sugarcane varieties. Nevertheless, it can be seen from Table 2 that the magnitude of the variability between the three sugarcane residues in terms of the components (cellulose, hemicellulose, lignin, and ash) was smaller than for the raw materials, suggesting that pretreatment with dilute sulfuric acid increased the similarity of the materials, irrespective of sugarcane variety. The highest cellulose and ash contents, as well as the lowest lignin contents, were found for the tops, while straw presented the lowest cellulose and hemicellulose contents and the highest lignin contents. Finally, bagasse showed the highest hemicellulose contents and the lowest ash contents. In order to observe more clearly the effect of dilute acid pretreatment on the sugarcane biomass, an order of chemical composition was established, before and after the pretreatment, in terms of the average contents of hemicellulose, lignin, cellulose, and ash for the different types of residue (bagasse, straw, and tops), as shown in Table 3. It is interesting to note that only the order of ash content remained unaltered after the dilute acid pretreatment.

**Table 3:** **Effect of the dilute sulfuric acid pretreatment on the order of the different residues**

**Component**	**Raw materials**	**Pretreated materials**
Cellulose	Straw > bagasse > tops	Tops > bagasse > straw
Hemicellulose	Tops > straw > bagasse	Bagasse > tops > straw
Lignin	Bagasse > straw > tops	Straw > bagasse > tops
Ash	Tops > straw > bagasse	Tops > straw > bagasse

Chemical composition orders based on average data for each type of residue (bagasse, straw, and tops) obtained for the four varieties (K, M, Q, and X).

The process of lignocellulosic ethanol production by means of bioconversion consists of three critical steps: pretreatment of the biomass, hydrolysis of sugar polymers to fermentable sugar monomers, and fermentation of sugar monomers to ethanol [40]. The pretreatment step is essential for effective ethanol production because of the natural recalcitrance of lignocellulosic biomass, which limits the access of enzymes to the structural matrix [41-44]. Among the various types of chemical biomass pretreatment, dilute acid is fast and is one of the oldest and most widely used methods, since it satisfies the majority of the requirements of pretreatment processes [10,45,46]. Lignocellulosic biomass submitted to dilute acid pretreatment undergoes partial hemicellulose solubilization and lignin redistribution [47]. As a result, the contents of cellulose and lignin are enhanced in the pretreated material, compared to the untreated biomass [21,24,26,34,48]. In the present study, the dilute acid pretreatment (H_2_SO_4_, 1.5% *w*/*w*, 1:10 solid/liquid ratio, 121°C, 30 min) provided effective enrichment of the biomass in terms of the cellulose and lignin contents, regardless of the residue type (bagasse, straw, or tops) or sugarcane variety (K, M, Q, or X). This was mainly achieved by removing the hemicellulosic fraction (Table 2).

The average contents of cellulose, hemicellulose, lignin, and ash found for the pretreated bagasse from the different sugarcane varieties (48.9 ± 1.3, 7.7 ± 0.1, 31.4 ± 1.0, and 0.6 ± 0.1% for varieties K, M, Q, and X, respectively) were slightly different to those reported in the literature using a dilute mixed acid pretreatment (1% *w*/*v* sulfuric acid and 1% *w*/*v* acetic acid solution, 1:10 solid/liquid ratio, 190°C, 10 min) [27] and comparable to a pretreatment employing dilute sulfuric acid (H_2_SO_4_, 2% *w*/*w*, 1/15 solid/liquid ratio, 150°C, 30 min) [47]. The results obtained here with respect to the chemical composition of sugarcane bagasse after the pretreatment with dilute acid (H_2_SO4, 1.5% *w*/*w*, 1:10 solid/liquid ratio, 121°C, 30 min) were similar to those reported previously using a solids loading of 15% (*w*/*w*) and a different cultivar of sugarcane [26].

As mentioned above, there have been few studies conducted to evaluate the chemical composition of sugarcane tops alone, and only hydrothermal pretreatment has been employed [32,37]. The same applies to the sugarcane trash, since there are few studies on the evaluation of its chemical composition in comparison to sugarcane bagasse. The effect of dilute acid pretreatment on hemicellulose solubilization was investigated in a recent study of the enzymatic hydrolysis of pretreated sugarcane trash [34]. Other pretreatments of sugarcane trash that have been studied are steam explosion [30,31] and hydrothermal [33] processes. Dilute acid, hydrothermal, and steam explosion pretreatments have all been extensively studied and used for cellulosic ethanol production, where they provide effective removal of the hemicellulosic fraction, opening up or improving the access of enzymes to the cellulosic fraction in the subsequent enzymatic hydrolysis step [49,50].

In earlier work, the efficiency of enzymatic hydrolysis of hydrothermally pretreated sugarcane trash (which includes dry leaves, green leaves, and tops) was investigated (1:10 solid/liquid ratio, 190°C, 10 min) and cellulose, hemicellulose, lignin, and ash contents of 51.6, 16.8, 30.0, and 1.4%, respectively, were reported for tops. The corresponding values for dry leaves (straw) were 53.9, 12.3, 29.5, and 3.5%, respectively [32]. The results of the dilute acid pretreatment (H_2_SO_4_, 1.5% *w*/*w*, 1:10 solid/liquid ratio, 121°C, 30 min) conducted here were superior in terms of the solubilization of hemicellulose, since the average contents of hemicellulose in the pretreated tops and straw were 7.5 ± 0.2 and 6.5 ± 0.3%, respectively.

### Enzymatic conversion of cellulose in the pretreated materials

**Figure 1:**
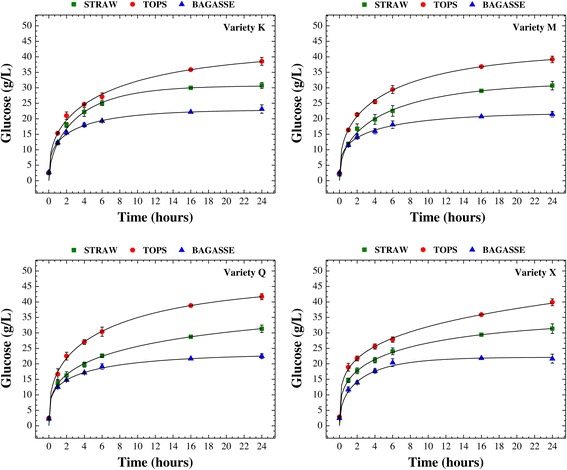
**Temporal profiles of glucose release.** Temporal profiles of glucose release during the enzymatic hydrolysis of bagasse, straw, and tops from different varieties of sugarcane (K, M, Q, and X) pretreated with dilute sulfuric acid. The lines are models fitted according to the Chrastil [61] approach.

Figure 1 presents the temporal profiles of glucose release during the enzymatic saccharification of bagasse, straw, and tops from the different varieties of sugarcane previously pretreated with dilute sulfuric acid (H_2_SO_4_, 1.5% *w*/*w*, 1:10 solid/liquid ratio, 121°C, 30 min). All the enzymatic hydrolysis assays were conducted under the same conditions to ensure the validity of comparisons. The enzymatic hydrolysis profiles showed similar patterns, regardless of sugarcane variety (K, M, Q, or X), as can be clearly seen in Figure 1. In this ethanol production step, the tops were found to be most susceptible to enzymatic degradation, with an average glucose concentration of 39.8 g/L reached after 24 h. The bagasse showed the lowest enzymatic digestibility, with 22.2 g/L glucose obtained in the same time. Glucose release from the tops was about 80% higher than from the bagasse. The straw provided an average of 31.0 g/L glucose, which was 40% higher compared to the bagasse and 28% lower in relation to the tops. An important finding was therefore that the qualitative order of sugar released from the sugarcane biomass was as follows: tops > straw > bagasse.

For a more precise analysis of the enzymatic hydrolysis step of the sugarcane lignocellulosic biomass pretreated with dilute acid, the data in terms of enzymatic conversion of cellulose are presented in Table 4. In an initial examination, the tops were most susceptible to enzymatic hydrolysis, followed by the straw and bagasse, with average values for cellulose conversion to glucose of 66.9, 51.8, and 35.5%, respectively. The sugarcane variety was not a significant factor in the enzymatic hydrolysis step, since the enzymatic conversion achieved for each type of residue (bagasse, straw, or tops) was statistically the same for all the sugarcane varieties (K, M, Q, and X), as revealed by Tukey’s test with a significance level of *P* < 0.05 (Table 4). In addition, the responses of the different residue types to saccharification were significantly different, corroborating the order of susceptibility to enzymatic conversion mentioned above (tops > straw > bagasse).

**Table 4:** **Enzymatic conversion of cellulose (ECC, %) after 24 h of hydrolysis**

**Variety**	**Symbol**	**Residue**	**Cellulose conversion (%)**
SP79-1011	K	Straw	52.53^B^
		Tops	65.54^A^
		Bagasse	35.82^C^
RB867515	M	Straw	51.68^B^
		Tops	67.45^A^
		Bagasse	35.45^C^
SP81-3250	Q	Straw	51.26^B^
		Tops	70.20^A^
		Bagasse	35.91^C^
RB92579	X	Straw	51.76^B^
		Tops	64.37^A^
		Bagasse	34.98^C^

Data refer to the hydrolysis of straw, bagasse, and tops. from four varieties of sugarcane (K, M, Q, and X) pretreated with dilute acid. Means with different subscript capital letters are statistically different (Tukey’s test, *P* < 0.05).

In work concerning the optimization of dilute sulfuric acid pretreatment to maximize the sugar yield (pentose and hexose sugars) from sugarcane bagasse for ethanol production, considerable variations in sugar yields from enzymatic hydrolysis were observed for different varieties of sugarcane [24]. In a subsequent study, the same authors reported that there were significant differences in the enzymatic digestibility of different varieties of sugarcane [23]. Different responses in the enzymatic hydrolysis step have similarly been reported for other varieties of sugarcane or alternative feedstocks such as switchgrass [36,47,51]. In this regard, the earlier findings differ from the results obtained here. However, it is important to consider the set of samples analyzed. It is likely that the sugarcane varieties employed here (SP79-1011, RB867515, SP81-3250, and RB92579) have similar physical-chemical-morphological characteristics. Accordingly, additional studies of these varieties of sugarcane are currently being conducted using spectrometric and microscopic techniques such as nuclear magnetic resonance, Fourier transform infrared spectroscopy, X-ray diffraction, and scanning electron microscopy, in order to elucidate these and other important issues.

The enzymatic hydrolysis of sugarcane bagasse and trash pretreated with dilute acid (H_2_SO_4_, 2.9% *w*/*v*, 1:4 solid/liquid ratio, 130°C, 30 min) was investigated by Moutta et al*.* [34]. The optimal conditions were established in a previous study [46]. In agreement with the present results, the sugarcane trash showed higher enzymatic digestibility than bagasse, and the authors suggested that it was due to the morphological characteristics and the distribution of structural macromolecules (cellulose, hemicellulose, and lignin) in the cell walls [34]. As result, cellulose was less accessible in the bagasse, which explained the lower yield of glucose than obtained using trash [34]. Interestingly, the aforementioned study employed loadings of enzymes per gram of biomass, while here, we used enzymatic loadings per gram of cellulose, with the aim of ensuring equal starting conditions for all the materials tested, since the enzyme/substrate ratio (E/S) has a large influence on the rate of the catalytic reaction. Another similar study from the same group assessed the effect of hydrothermal pretreatment (1:10 solid/liquid ratio, 195°C, 10 min) on the enzymatic conversion of bagasse and trash, and reported that after 24 h, the glucose concentrations from the pretreated trash and bagasse reached 17.2 and 13.5 g/L, corresponding to cellulose hydrolysis yields of 84.4 and 49.1%, respectively [33]. These findings emphasize the lower susceptibility of bagasse to enzymatic hydrolysis, compared to the trash (straw and tops), irrespective of whether the pretreatment is performed using dilute acid or a hydrothermal process.

An important point is that lignin is considered the most recalcitrant component of the plant cell wall macromolecules. It is predominantly found in the secondary cell wall and plays a key role in pathogen resistance, water regulation, and maintaining the integrity of the cell wall structure [50]. The relation between lignin content and enzymatic degradation of vegetal biomass has been extensively studied in recent years [36,52,53]. In general, it is reported that the lower the lignin content, the greater will be the bioavailability of the substrate for the production of ethanol [50]. Here, all the sugarcane biomass parts showed similar lignin contents after the pretreatment step (Table 2), with the following order for this component: straw > bagasse > tops (Table 3). Therefore, the lower susceptibility to enzymatic conversion observed here for bagasse is not only related to the lignin content, and other factors related to the physical-chemical characteristics of the biomass could also play a role, as further discussed in the following sections.

### Cellulosic hydrolysate fermentability

**Figure 2:**
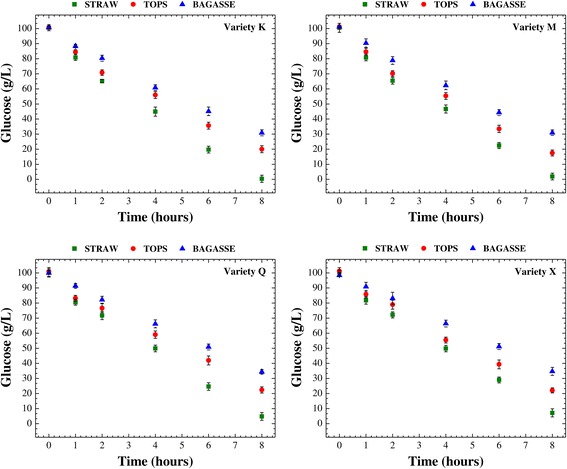
**Temporal profiles of glucose consumption.** Temporal profiles of glucose consumption throughout the alcoholic fermentation of the cellulosic hydrolysates resulting from the enzymatic saccharification of bagasse, straw, and tops from different varieties of sugarcane (K, M, Q, and X) pretreated with dilute sulfuric acid.

**Figure 3:**
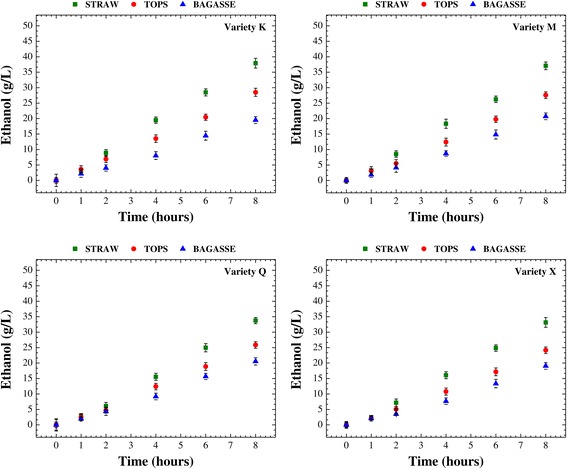
**Temporal profiles of ethanol production.** Temporal profiles of ethanol production during the course of the alcoholic fermentation of the cellulosic hydrolysates from the enzymatic hydrolysis of bagasse, straw, and tops from different varieties of sugarcane (K, M, Q, and X) pretreated with dilute sulfuric acid.

After production of the cellulosic hydrolysates, alcoholic fermentation was performed in order to evaluate the fermentability of these media. The fermentation step followed the same conditions for all assays, in order to ensure the same basis for comparison of the individual responses for each type of sugarcane residue (bagasse, straw, or tops) from the four sugarcane varieties. Figures 2 and 3 present the temporal profiles of glucose consumption and ethanol production, respectively, throughout 8 h of alcoholic fermentation employing an industrial strain of *Saccharomyces cerevisiae*. Overall, the alcoholic fermentation profiles (glucose consumption and ethanol production) showed similar patterns, regardless of sugarcane variety (Figures 2 and 3). In this step of the cellulosic ethanol production process, the hydrolysates obtained from the straw of all varieties provided the best fermentability, consuming on average more than 95% of the available glucose (Figure 2) and reaching an average ethanol concentration of 35.5 g/L after 8 h (Figure 3). The lowest fermentability was found for the media derived from the bagasse of all varieties, with consumption of slightly more than 65% of the glucose (Figure 2) and production of 20.0 g/L ethanol (Figure 3). Ethanol production was about 78% higher for the hydrolysates from straw, compared to those from bagasse. The results obtained for the hydrolysates from the tops were intermediate between those for straw and bagasse, with consumption of around 80% of the glucose (Figure 2) and production of 26.5 g/L ethanol (Figure 3), corresponding to an ethanol production that was 33% higher compared to the hydrolysates from bagasse and 34% lower in relation to those from straw. The qualitative order of fermentability for the hydrolysates from the different sugarcane residues was therefore as follows: straw > tops > bagasse.

Determination of the ethanol yield (% of theoretical) provides essential information for evaluation of the ethanol production process, as shown in Table 5. The responses of each type of hydrolysate (from bagasse, straw, or tops) to alcoholic fermentation showed statistically significant differences, with the hydrolysates produced from straw being most fermentable, followed by those from tops and bagasse, with average ethanol yields of 74.1, 65.1, and 56.3%, respectively (Table 5). Statistical analysis indicated that the variety of sugarcane was not a significant factor in the alcoholic fermentation step, with the ethanol yields for each individual type of hydrolysate (from bagasse, straw, or tops) being statistically the same (Tukey’s test, *P* < 0.05) for all the varieties (Table 5).

**Table 5:** **Ethanol yield (% of theoretical) after 8 h of alcoholic fermentation**

**Variety**	**Symbol**	**Residue**	**Ethanol yield (%)**
SP79-1011	K	Straw	74.70^A^
		Tops	65.52^B^
		Bagasse	54.69^C^
RB867515	M	Straw	74.19^A^
		Tops	64.47^B^
		Bagasse	55.91^C^
SP81-3250	Q	Straw	73.90^A^
		Tops	63.85^B^
		Bagasse	58.16^C^
RB92579	X	Straw	73.74^A^
		Tops	66.38^B^
		Bagasse	56.30^C^

Data are from the cellulosic hydrolysates resulting from the enzymatic hydrolysis of straw, tops, and bagasse from four varieties of sugarcane (K, M, Q, and X) pretreated with dilute acid.

Means with different subscript capital letters are significantly different (Tukey’s test, *P* < 0.05).

The influence of variety on cellulosic ethanol production has been evaluated for different feedstocks such as sugarcane, poplar, and wheat [22,23,36,47,51,54,55]. It has generally been found that different varieties of the same raw material differ in terms of overall ethanol production, suggesting that the selection step should identify varieties requiring minimal pretreatment and enzymatic hydrolysis steps, which would reduce the total cost of the process. For example, use of varieties of sugarcane bagasse with lower lignin content and highly substituted xylan resulted in higher sugar and ethanol yields, with milder pretreatment conditions and reduced enzyme dosage [23]. On the other hand, the results found here concerning the influence of sugarcane variety on the fermentability of the hydrolysates revealed no significant differences in the ethanol yield. As mentioned before, this could have been due to the similar physical-chemical-morphological characteristics of the set of sugarcane varieties evaluated here.

The commercial sugarcane varieties employed (SP79-1011, RB867515, SP81-3250, and RB92579) are among the most widely cultivated in central-south Brazil [56] and show similar agronomic characteristics including low water demand, good mechanical harvesting, and efficient transportation. Differences between the varieties are the greater soil requirement of SP79-1011, later maturation of RB867515, and poorer drought tolerance of SP81-3250 and RB92579. Acknowledging that the set of varieties evaluated here was relatively small, the variety of sugarcane was not a factor that significantly influenced the cellulosic ethanol production process. Selection of variety for cultivation should therefore focus on agronomic aspects such as adaptation to regional soil and weather conditions, in order to maximize productivity and yield while minimizing management costs. Furthermore, this finding is an important indication of the possibility of cultivating different varieties of sugarcane without affecting the final production of cellulosic ethanol in the mills. It is vital that the producer has a diversity of sugarcane varieties in the field, in order to decrease the likelihood of damage due to proliferation of pests and diseases within the plantation [56].

Another important fermentation parameter is the volumetric ethanol productivity (*Q*_P_). The highest productivities were achieved for the hydrolysates from straw (*Q*_P_ = 4.4 g/L.h), followed by those from tops (*Q*_P_ = 3.3 g/L.h) and bagasse (*Q*_P_ = 2.5 g/L.h). These values were averages for each type of hydrolysate (from bagasse, straw, or tops) after 8 h of alcoholic fermentation and were statistically different. Therefore, the volumetric ethanol productivity for bagasse hydrolysates was about 76% lower compared to straw hydrolysates and approximately 32% lower in relation to tops hydrolysates. This can be explained by the negative effect of aromatic substances (mainly phenolic compounds) on volumetric ethanol productivity. These substances can be released from residual lignin and interfere in the substrate assimilation rate, hence affecting ethanol productivity [47].

In terms of alcoholic fermentation, another essential aspect concerns the inhibition of microorganisms, especially given the known inhibition of ethanol-producing yeast by degradation products produced during pretreatment of biomass. The generation of pretreatment by-products is strongly dependent on the feedstock and pretreatment method. Dilute sulfuric acid pretreatment is recognized to produce by-products considered inhibitory to microbial fermentation [10], such as furan derivatives, weak acids, and phenolic compounds [57,58]. The main furan derivatives are furfural and 5-hydroxymethyl furfural (HMF) derived from degradation of pentoses and hexoses, respectively, while weak acids such as acetic acid are formed from the acetic groups present in the hemicellulosic fraction [41].

The temporal profiles of glucose consumption (Figure 2) and ethanol production (Figure 3) during the alcoholic fermentation of the hydrolysates (from bagasse, straw, or tops) clearly showed that the presence of inhibitory substances could have interfered in the metabolism of the yeast strain used in this study. Since there were no significant differences among the varieties of sugarcane evaluated here in terms of the overall process of ethanol production, measurements of inhibitors (acetic acid, HMF, and furfural) were only made during alcoholic fermentation of the hydrolysates (from bagasse, straw, and tops) obtained using the variety K. No detectable levels of furfural were found in any of the hydrolysates after 8 h of alcoholic fermentation. On the other hand, very low levels of acetic acid and HMF were detected, with the highest amounts in the hydrolysate from bagasse (481.8 and 12.2 mg/L, respectively) and the lowest concentrations in the hydrolysate from straw (154.7 and 10.2 mg/L, respectively). The hydrolysate from tops presented intermediate levels of both inhibitors (318.5 mg/L of acetic acid and 11.5 mg/L of HMF). These findings were in correlation with the order of fermentability described above (straw > tops > bagasse).

It has been found previously that after milling and pretreatment of sugarcane bagasse and trash, the bagasse hydrolysates contained higher levels of acetic acid, compared to the trash hydrolysates, resulting in an ethanol yield that was somewhat higher when trash hydrolysates were used [35], supporting the results found here. The present findings obtained from the assessment of separate sugarcane residues therefore clearly showed that ethanol production from sugarcane biomass could be improved by the combined use of bagasse, straw, and tops. This approach is an interesting alternative because use of the whole sugarcane lignocellulosic biomass could greatly improve ethanol productivity per hectare, without any need to expand the areas under cultivation. To the best of our knowledge, there have been no previous studies of this strategy including all the steps of cellulosic ethanol production.

### Combined use of the whole sugarcane lignocellulosic biomass for ethanol production

**Figure 4:**
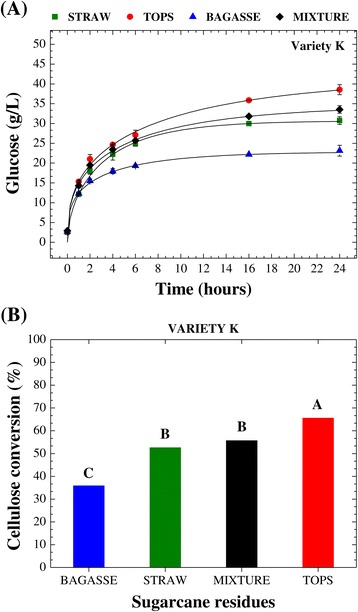
**Temporal profiles of glucose release and statistical analysis of the data for enzymatic conversion.** Comparison of the temporal profiles of glucose release **(A)** and statistical analysis of the data for enzymatic conversion of cellulose after 24 h **(B)** for the saccharification of bagasse, straw, tops, and the combination of them (bagasse-straw-tops, 1:1:1 mixture), using variety K. The lines are models fitted according to the Chrastil [61] approach. Means with different capital letters are statistically different (Tukey’s test, *P* < 0.05).

After assessing the responses for each type of residue (bagasse, straw, and tops) obtained after processing of the four sugarcane varieties (K, M, Q, and X), investigation was made of the feasibility of using a mixture of the three sugarcane residues for the conversion of cellulose into glucose and ethanol production. For this purpose, variety K was arbitrarily selected, since there were no significant differences among the varieties in terms of enzymatic conversion (Table 4). Figure 4A shows the temporal profiles of glucose release during the enzymatic hydrolysis step for the three separate residues and a mixture of them (bagasse-straw-tops, 1:1:1, dry weight basis). The chemical composition of the pretreated mixture was calculated as a weighted average of the compositions of the individual pretreated residues.

As expected, the enzymatic saccharification of the mixture showed a pattern that was intermediate to those for the individual residues (Figure 4A). Figure 4B presents the data for the enzymatic conversion of cellulose in these processes, together with a statistical analysis (Tukey’s test, *P* < 0.05). The conversion value obtained for the mixture was significantly higher than that for bagasse and significantly lower than that for the tops. The conversion value for the mixture was statistically the same as that for the straw. These results are encouraging in terms of the use of whole sugarcane lignocellulosic biomass because they show that enzymatic saccharification can be performed using the residues from the processing of sugarcane together in a mixture (bagasse-straw-tops). In this biotechnological approach, the lower susceptibility to enzymatic degradation of bagasse can be balanced by the higher susceptibility of straw and tops (trash), as can be seen in Figure 4A,B. Importantly, the enzymatic conversion obtained using the mixture was 55% higher, compared to the use of bagasse alone. Therefore, the potential application of bagasse (which is already being studied) could be further extended if combined with the use of tops and straw.

In previous work, the influence of biomass chemical composition on enzymatic conversion of cellulose into glucose has been evaluated by correlating the yield of glucose with the contents of lignin, hemicellulose, and ash. No direct relation between residual lignin or hemicellulose and the enzymatic conversion of cellulose was observed for bagasse derived from different sugarcane hybrids [47]. Nevertheless, the lignin/hemicellulose (L/H) ratio showed a strong correlation with conversion yield (*R*^2^ = 0.90), with maximum conversion values achieved when the L/H ratio was in the range from 3 to 4 [47]. On the other hand, several other studies have reported negative correlations between cellulose digestibility and the lignin and ash contents [21,22,24,36,55]. Nonetheless, the enzymatic conversion of biomass into glucose is not exclusively determined by the lignin and ash contents, but it also depends on the contents of structural carbohydrates as well as other physical and chemical properties of these lignocellulosic materials [55]. Moreover, even though lignin is usually assumed to be one of the most important factors limiting aspects of the saccharification, the presence of residual hemicellulose cannot be ignored, because its close association with the cellulose fibrils hinders access of the cellulases to the cellulose surface [47].

**Figure 5:**
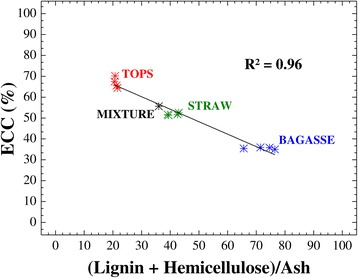
**Negative linear correlation between the enzymatic conversion of cellulose and the (lignin + hemicellulose)/ash ratio.**

Here, no direct correlations were observed between cellulose conversion and the lignin or hemicellulose contents of the pretreated materials, and there was no relation between conversion and the L/H ratio. However, there was a strong negative correlation (*R*^2^ = 0.96) between the enzymatic conversion values for the residues (bagasse, straw, and tops) from the four sugarcane varieties and the (lignin + hemicellulose)/ash ratio (Figure 5). Hence, the highest enzymatic conversion was achieved when this ratio was smallest. This correlation was able to explain the observed order of enzymatic digestibility (tops > straw > bagasse), since the three sugarcane residues obtained from all varieties were distributed in three independent groups. Inclusion of the conversion value obtained for the mixture of the three residues (bagasse-straw-tops, 1:1:1, dry weight basis) showed that the enzymatic conversion of the whole sugarcane lignocellulosic biomass into glucose could be correlated with the (lignin + hemicellulose)/ash ratio (Figure 5). Since statistical analysis showed that there was no significant difference between the straw and the mixture, in terms of the conversion of cellulose (Figure 4B), the influence of the chemical composition of the materials was not related to the individual components, but rather to the ratios between them, indicating the importance of the distribution of the different components.

**Figure 6:**
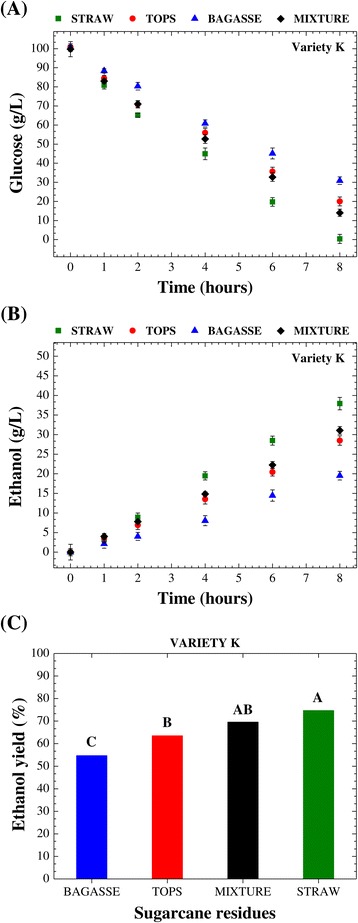
**Temporal profiles of glucose consumption and ethanol production and the data for the ethanol yield.** Comparison of the temporal profiles of glucose consumption **(A)** and ethanol production **(B)**, and statistical analysis of the data for the ethanol yield (% of the theoretical yield) after 8 h **(C)** for the alcoholic fermentation of the cellulosic hydrolysates from bagasse, straw, tops, and the combination of them (bagasse-straw-tops, 1:1:1 mixture), using variety K. Means with different capital letters are statistically different (Tukey’s test, *P* < 0.05).

After individual assessment of the different hydrolysates (from bagasse, straw, and tops) in terms of their fermentability, evaluation was made of the fermentation behavior of a hydrolysate from enzymatic hydrolysis of a mixture of the three sugarcane residues (bagasse-straw-tops, 1:1:1, dry weight basis) pretreated with dilute sulfuric acid. Figure 6 presents the temporal profiles of glucose consumption (A) and ethanol production (B). In this phase of the study, it was important to examine if the fermentation of a hydrolysate generated after enzymatic conversion of a sugarcane residues mixture would show any unexpected effects, or even be detrimental to the production of ethanol. Since there were no significant differences among the varieties in terms of alcoholic fermentation (Table 5), variety K was again arbitrarily selected for this comparison. The alcoholic fermentation of hydrolysate from the mixture (bagasse-straw-tops, 1:1:1, dry weight basis) exhibited an intermediate pattern, compared to the hydrolysates from bagasse, straw, and tops, as can be clearly seen from the data for glucose consumption (Figure 6A) and ethanol production (Figure 6B). The fermentation of hydrolysate from the mixture was monitored in terms of the contents of furfural, HMF, and acetic acid. Again, there were no detectable levels of furfural. The acetic acid and HMF levels were intermediate (267.6 and 10.8 mg/L, respectively) compared to the hydrolysates from bagasse, straw, and tops. Furthermore, statistical analysis (Tukey’s test, *P* < 0.05) of the ethanol yield data for these fermentation processes showed that there were no significant differences between the hydrolysates from the mixture and from tops or straw (Figure 6C). On the other hand, the ethanol yield value for the mixture was significantly higher than for bagasse. The volumetric productivity of ethanol for the hydrolysate from the mixture was also calculated (*Q*_P_ = 3.9 g/L.h) and was approximately 56% higher compared to the productivity of ethanol for the hydrolysate from bagasse (*Q*_P_ = 2.5 g/L.h).

An important point is that the yield of ethanol found for the hydrolysate from the mixture was about 25% higher than for the hydrolysate from bagasse (Figure 6C). These results are highly encouraging for the strategy of using the whole sugarcane plant for the production of cellulosic ethanol, since the fermentability of hydrolysates obtained from the enzymatic hydrolysis of individual sugarcane residues was comparable to that for a mixture of them (bagasse-straw-tops, 1:1:1, dry weight basis), so use of the mixture had no negative effects on the overall process. Thus, the lower fermentability presented by the hydrolysate from bagasse could be balanced by higher fermentability found for the hydrolysates from straw and tops (trash), as shown in Figure 6. It can be concluded that overall cellulosic ethanol production can be increased by using a mixture of the whole sugarcane lignocellulosic biomass (bagasse, straw, and tops), instead of bagasse alone.

**Figure 7:**
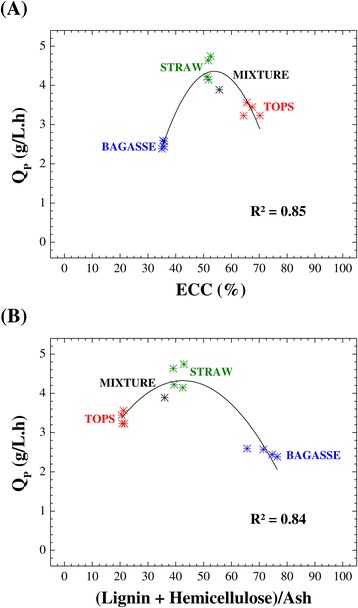
**Ethanol productivity and cellulose conversion and chemical composition of the pretreated biomass.** Relationship between volumetric ethanol productivity and enzymatic conversion of cellulose **(A)**, and relationship between the volumetric ethanol productivity and the (lignin + hemicellulose)/ash ratio **(B)**.

In order to explain how the chemical composition of biomass could affect the production of ethanol, it was reported previously that the ethanol concentration presented a strong inverse correlation (*R*^2^ = 0.91 to 0.99) with lignin content [23]. In the present study, there were no direct correlations between ethanol yield (or volumetric productivity of ethanol) and the residual contents of lignin or hemicellulose from the pretreated materials or between the ethanol yield and the L/H ratio. On the other hand, it is expected that there is a positive correlation between the glucose yield in the enzymatic saccharification and the ethanol yield from the alcoholic fermentation, that is, an increase in the enzymatic conversion of cellulose into glucose will result in greater amount of fermentable sugar readily available for the ethanol production step. Here, the best cellulose conversions were obtained for the tops from the different varieties, whose hydrolysates resulted in intermediate volumetric ethanol productivity values (or ethanol yields). The highest fermentability values were obtained for the hydrolysates from straw. In order to better understand these observations, the volumetric productivity of ethanol (*Q*_P_, g/L.h) and the enzymatic conversion of cellulose (ECC, %) were examined for each hydrolysate type (bagasse, straw, and tops) from the four sugarcane varieties. A meaningful relationship (*R*^2^ = 0.85) was found between *Q*_P_ and ECC, which could be fitted using a second-order polynomial (Figure 7A). As mentioned previously, a strong inverse linear correlation was established between the conversion of cellulose (ECC, %) and a ratio involving the contents of lignin, hemicellulose, and ash (Figure 5). Investigation of the influence of the latter on ethanol productivity revealed a strong relationship (*R*^2^ = 0.84), and a good fit was obtained using a second-order polynomial (Figure 7B).

The relationships illustrated in Figure 7A,B provided a qualitative order of fermentability, straw > tops > bagasse, with all the sugarcane varieties contained in three distinct groups corresponding to the three residues. The value for the mixture of the three residues (bagasse-straw-tops, 1:1:1, dry weight basis) was in agreement with the polynomial fit, showing that the ethanol productivity could be related to both the cellulose conversion and the chemical composition of the pretreated biomass (Figure 7). A possible explanation for the fact that the greater susceptibility to enzymatic degradation displayed by the tops did not lead to a higher yield of ethanol for the hydrolysates from the tops could have been due to a greater propensity for generation of degradation products. This was supported by the presence of inhibitory substances (HMF and acetic acid) at higher concentrations in the hydrolysates from the tops, compared to the hydrolysates from the straw (see the previous section). The greater potential of the sugarcane tops to produce degradation products, compared to the straw, seems to be closely related to the chemical composition of these lignocellulosic materials. These factors could help to explain the interesting trends illustrated in Figure 7A,B. Notwithstanding, this promising biotechnological approach, employing the whole sugarcane lignocellulosic biomass for ethanol production, can help in overcoming the difficulties experienced in the enzymatic hydrolysis and alcoholic fermentation steps using bagasse alone because the mixture of sugarcane residues (bagasse-straw-tops) provides a means of compensating for the lower degradability and fermentability of bagasse.

## Conclusions

Within the context of the current trend towards using the whole sugarcane lignocellulosic biomass, the lignocellulosic residues (bagasse, straw, and tops) from four varieties of sugarcane (SP79-1011, RB867515, SP81-3250, and RB92579) were assessed for the production of ethanol, under the same conditions. The chemical compositions of the residues pretreated with dilute sulfuric acid showed only slight differences, indicating that the pretreatment was able to produce similar contents of the main components (cellulose, hemicellulose, lignin, and ash) in the three types of pretreated residue. Susceptibility to the enzymatic saccharification step was in the order: tops > straw > bagasse. For the alcoholic fermentation step, the fermentability of the hydrolysates from the different sugarcane residues was in the order: straw > tops > bagasse. Sugarcane variety was not a significant factor in the overall ethanol production process. The proposed strategy is a promising way in which the whole sugarcane lignocellulosic biomass can be used in combination for the production of cellulosic ethanol. The use of the mixture (bagasse-straw-tops, 1:1:1, dry weight basis) resulted in an intermediate pattern, compared to the residues used individually, without any notable deleterious effects. In addition, the enzymatic conversion of cellulose obtained with the mixture was about 55% higher than for the bagasse alone. Similarly, the ethanol yield for the hydrolysate derived from the mixture was 25% higher than obtained using bagasse alone. These findings show that the lower susceptibility of sugarcane bagasse to enzymatic degradation, as well as the lower fermentability of the hydrolysates from sugarcane bagasse, can be assisted by using the bagasse in combination with the tops and straw (sugarcane trash). The use of bagasse, which is already being widely studied as a feedstock for production of 2G ethanol, can be further extended by employing the whole sugarcane lignocellulosic biomass.

## Materials and methods

### Raw materials and biomass preparation

Three lignocellulosic residue parts (bagasse, straw, and tops) from four varieties of sugarcane (SP79-1011, RB867515, SP81-3250, and RB92579, here represented by K, M, Q and X, respectively) were kindly supplied by Sumaúma Mill (Marechal Deodoro, Brazil). These lignocellulosic materials were dried in an oven at 45°C until reaching moisture content below 10%. The samples were then milled in a knife mill, sieved at 2 mm, and stored at room temperature for later use. The raw materials were chemically characterized (on a dry matter basis) in terms of their contents of structural carbohydrates (cellulose and hemicellulose), lignin, and ash according to Gouveia et al*.* [59].

### Dilute sulfuric acid pretreatment

The pretreatment of the lignocellulosic materials was carried out by a chemical pretreatment method using dilute acid. This process employed a solution of dilute sulfuric acid (1.5%, *w*/*w*) and a solid loading of 10%. The reactions were performed at 121°C for 30 min in an autoclave. After the pretreatment, the liquid and solid fractions (hemicellulosic hydrolysates and cellulignins, respectively) were separated by vacuum filtration. The hemicellulosic hydrolysates were discarded. The pretreated solid materials were thoroughly washed with distilled water to remove the soluble components and used directly for the next steps. To assess the combination of the residues, a mixture of bagasse, straw, and tops (1:1:1, dry weight basis) was prepared and pretreated following the same procedure described previously. The pretreated materials were also chemically characterized according to Gouveia et al*.* [59].

### Enzymatic hydrolysis step

A commercial enzyme preparation (Cellic®Ctec2), kindly provided by Novozymes A/S (Bagsværd, Denmark), was used for the enzymatic hydrolysis assays of the pretreated materials. The enzymatic activity of commercial extract, in terms of filter paper units (FPU), was determined according to Ghose [60]. The enzymatic hydrolyses of the pretreated materials were performed in 500-mL Erlenmeyer flasks containing 0.1 M sodium citrate buffer (pH 5.0), at a solid/liquid ratio of 1:10 and using an enzyme loading of 30 FPU per gram of residual cellulose in the pretreated materials. The reaction mixtures were incubated for 24 h at 50°C and 200 rpm. The saccharification of the mixture (bagasse-straw-tops, 1:1:1, dry weight basis) was carried out under the same conditions described above. Samples were periodically withdrawn (0, 1, 2, 4, 6, and 24 h) for quantification of glucose release using an enzymatic kit Doles® (Goiânia, Brazil). In addition, in order to simulate the sampling after 16 h, the Chrastil approach for modeling enzymatic reactions was used to fit the experimental data for glucose release [61].

### Alcoholic fermentation step

An industrial strain of *S. cerevisiae* CAT-1 acquired from the Jalles Machado Mill (Goianésia, Brazil) was used for the alcoholic fermentation assays of the cellulosic hydrolysates, carried out in 250 mL Erlenmeyer flasks. After the saccharification step, the residual solid was separated by centrifugation. The levels of glucose in the cellulosic hydrolysates were measured, and anhydrous glucose was added up to a concentration of 100 g/L. The media were then inoculated with 25 g of yeast cells (on dry weight basis) per liter of cellulosic hydrolysate. The experiments were conducted for 8 h using a shaker at 31°C and 100 rpm. For the cellulosic hydrolysates from the enzymatic hydrolysis of the mixture (bagasse-straw-tops, 1:1:1, dry weight basis), the fermentation step was conducted under the same conditions described above. Throughout the alcoholic fermentation, the consumption of glucose was followed using an enzymatic kit Doles® (Goiânia, Brazil) and ethanol was monitored by HPLC (see next section).

### Analytical methods

The concentrations of monomeric sugars (cellobiose, glucose, xylose, and arabinose), organic acids (formic and acetic), furans (furfural and hydroxymethylfurfural), and ethanol were determined by high-performance liquid chromatography (HPLC) according to Gouveia et al. [59]. The determination of monomeric sugars, organic acids, and ethanol used a refractive index detector and an Aminex HPX-87H column. The chromatographic separation was conducted using isocratic elution with 5 mM sulfuric acid as the mobile phase, at a flow rate of 0.6 mL/min, and oven and detector cell temperatures of 50°C. Quantification of the furans employed a UV–vis detector (274 nm) and an Agilent Sorbex C18 column (250 × 2.5 mm, particle size 5 μm). The chromatographic separation was conducted using isocratic elution, with a solution of acetonitrile/water (1:8) containing 1% (*v*/*v*) acetic acid as the mobile phase, at a flow rate of 0.8 mL/min, and an oven temperature of 25°C.

### Data and statistical analysis

All the experimental steps (chemical characterization, dilute acid pretreatment, enzymatic hydrolysis, and alcoholic fermentation) were carried out in triplicate, and the results were calculated as means ± standard deviations. In all cases, the standard deviations were less than 5%. Thus, only the mean values are presented in the Tables. The error bars shown in the figures represent the standard deviations for triplicate assays, and where error bars are not visible, they are smaller than the symbol. The mean values were analyzed statistically using Tukey’s test with a confidence level of 95% (Origin 8.0 software).

### Calculations

For the enzymatic saccharification step, the enzymatic conversion of cellulose (ECC, %) was calculated according to Equation 1:

1 $$ \mathrm{E}\mathrm{C}\mathrm{C}=\left[\frac{\left({m}_{\mathrm{glucose}}^{24\mathrm{h}}-{m}_{\mathrm{glucose}}^{0\mathrm{h}}\right)}{\left({m}_{\mathrm{cellulose}}^{0\mathrm{h}}\right)*1.11}\right]*100 $$

For the alcoholic fermentation step, three parameters were calculated: the ethanol yield factor (*Y*_P/S_), described by Equation 2, which was used to calculate the ethanol yield (% of the theoretical yield), according to Equation 3, and the volumetric ethanol productivity (*Q*_P_, g/L.h), described by Equation 4.

2 $$ {Y}_{P/S}=\frac{\left({C}_{\mathrm{ethanol}}^{8\mathrm{h}}-{C}_{\mathrm{ethanol}}^{0\mathrm{h}}\right)}{\left({C}_{\mathrm{glucose}}^{0\mathrm{h}}-{C}_{\mathrm{glucose}}^{8\mathrm{h}}\right)} $$

3 $$ \mathrm{Ethanolyield}=\left(\frac{Y_{P/S}}{0.511}\right)*100 $$

4 $$ {Q}_P=\frac{\left({C}_{\mathrm{ethanol}}^{8\mathrm{h}}-{C}_{\mathrm{ethanol}}^{0\mathrm{h}}\right)}{8\mathrm{h}\hbox{-} 0\mathrm{h}} $$

In the above equations, *m* is the mass and *C* is the concentration of the compounds. The value 1.11 is the theoretical yield factor for the enzymatic conversion of cellulose to glucose (Equation 1). Likewise, 0.511 is the theoretical yield factor for the alcoholic fermentation process. In some studies, the ethanol yield (Equation 3) is sometimes called the fermentation efficiency.

## Abbreviations

2G: second generation
1G: first generation
db: dry basis
ECC: enzymatic conversion of cellulose
FPU: filter paper units
GHG: global greenhouse gas
HMF: 5-hydroxymethyl furfural
HPLC: high-performance liquid chromatography
*Q*_P_: ethanol productivity
*Y*_P/S_: ethanol yield factor
